# Ti_3_C_2_T*_x_* MXene-Based Light-Responsive Hydrogel Composite for Bendable Bilayer Photoactuator

**DOI:** 10.3390/nano10071419

**Published:** 2020-07-21

**Authors:** Sifani Zavahir, Patrik Sobolčiak, Igor Krupa, Dong Suk Han, Jan Tkac, Peter Kasak

**Affiliations:** 1 Center for Advanced Materials, Qatar University, P.O. Box 2713 Doha, Qatar; fathima.z@qu.edu.qa (S.Z.); patrik@qu.edu.qa (P.S.); igor.krupa@qu.edu.qa (I.K.); dhan@qu.edu.qa (D.S.H.); 2 Institute of Chemistry, Slovak Academy of Sciences, Dubravska cesta 9, 84538 Bratislava, Slovakia; jan.tkac@savba.sk

**Keywords:** MXene, photoactuator, NIPAm, bilayer hydrogel

## Abstract

Soft actuators based on hydrogel materials, which can convert light energy directly into mechanical energy, are of the utmost importance, especially with enhancements in device development. However, the hunt for specific photothermal nanomaterials with distinct performance remains challenging. In this study, we successfully fabricated a bilayer hydrogel actuator consisting of an active photothermal layer from incorporated Ti_3_C_2_T_*x*_ MXene in poly(N-isopropylacrylamide) p(NIPAm)hydrogel structure and a passive layer from the N-(2-hydroxylethylpropyl)acrylamide (HEAA) hydrogel structure. The uniform and effective incorporation of MXene into the NIPAm hydrogel structures were characterized by a battery of techniques. The light responsive swelling properties of the MXene-embedded NIPAm-based hydrogel demonstrated fully reversible and repeatable behavior in the light on–off regime for up to ten consecutive cycles. The effect of MXene loading, the shape of the actuator, and the light source effects on the bilayer NIPAm-HEAA hydrogel structure were investigated. The bilayer hydrogel with MXene loading of 0.3% in the NIPAm hydrogel exhibited a 200% change of the bending angle in terms of its bidirectional shape/volume after 100 s exposure to white light at an intensity of 70 mW cm^−2^. Additionally, the bending behavior under real sunlight was evaluated, showing the material’s potential applicability in practical environments.

## 1. Introduction

Actuators show changes in their behaviors, shapes, structures, or properties upon exposure to external stimuli such as heat [1,2], light [3,4], pH [5,6], ionic strength [7], and magnetic or electric fields [8]. Materials based on the piezoelectric effect, hydrogels, and fluid flows are widely studied actuator materials. Among these materials, hydrogels have gained much attention because of their high versatility, softness, wetting, and ability to be tailored to specific properties [9,10,11]. Hydrogels consist of water in water-insoluble, highly hydrophilic three-dimensional polymeric networks containing physical and/or chemical crosslinking [12]. Hydrogel actuators (or so-called smart hydrogel materials), upon being stimulated by hydrogels, lead to an adjustable sol–gel transition that causes a change in swelling that is transformed into an alteration in shape or volume [13,14]. One of the most commonly studied and recognized synthetic hydrogel materials is based on poly(N-isopropylacrylamide) p(NIPAm).

Hydrogels prepared using the NIPAm-based design are widely applied and studied in thermoresponsive materials [15]. Different architectures and designs are used for drug delivery, tissue engineering, sensing, and others [16,17,18]. Hydrogel materials from NIPAm show a lower critical solution temperature (LCST) (close to human body temperature) of 32 °C. LCST materials lead to a transition from a coil to a globule when the polymeric NIPAm network is changed from a swelled state below the LCST (driven by hydrogen bonding of the monomer units with the water molecules) to a shrinking state with phase separation above the LCST that is driven by the entropic factor.

Apart from the thermoresponsive character of NIPAm-based material, the incorporation of material enables light to undergo thermal conversion due to the material’s photothermal behavior [19]. Light as a stimulus offers an interesting platform for studying switch-like on–off behavior based on spatial location and time. Moreover, changes in behavior under different wavelengths of light can be tuned. In some studies, carbon nanotube- [20], graphene- [21,22], and magnetite-based [23] structures are incorporated into the hydrogel or polymer matrix to facilitate sufficient light-to-heat conversion to effectively improve light induced volume changes. There is still enormous effort needed to find a good candidate for conversion and application as a new nanomaterial. Analogous to the GO in the layer dimension, the role and subsequent performance of carbide-based 2D MXene in photoactuator systems is worth studying.

MXene is a relatively new type of 2D material derived from the MAX phase (two-dimensional closely packed layered structure of M transition metal, A is an A-group element and X is C and/or N) etching of the A phase by a selective method [24,25]. Such materials possess interesting properties such as high electrical conductivity and thermal stability, hydrophilic characteristics, and a high surface area [26]. These materials have applications mainly in energy storage [27,28], sensing [29], electromagnetic shielding [30,31], water technologies [32], and biomedicine [33,34]. The most commonly studied and accessible is Ti_3_C_2_T*_x_*, which is one of the most promising materials for photothermal conversion. It was shown that MXene’s light-to-heat conversion efficiency for Ti_3_C_2_T*_x_* can reach up to 100%. Further, the membrane from Ti_3_C_2_T*_x_*, with a heat barrier, offers a light-to-water evaporation efficiency of 84% under one sun irradiation [35]. Moreover, the material provides strong optical absorption in the near-IR (NIR) region with potential as a photothermal agent for tumor therapy [36,37,38]. Considering the photothermal efficiency of the nanomaterial properties of MXene, we focused on the fabrication process [39] and application of nanocomposite hydrogels possessing Ti_3_C_2_T*_x_* MXene [40,41] to exploit the volume shrinkage promoted by light in NIPAm-based hydrogel materials [42,43].

In this study, well-dispersed MXene was embedded into a NIPAm-based hydrogel network with photothermal behavior and induced transition. The nanocomposites exhibited robust and fast light- responsive behavior with reversible characteristics that were documented over ten consecutive cycles. Furthermore, the fabrication process for the hydrogel bilayer photoactuator was developed to construct the bilayer-based bending actuator. The actuation of different shapes was evaluated, as was sunlight actuation.

## 2. Materials and Methods

### 2.1. Materials

N-isopropylacrylamide (NIPAm), N-(2-hydroxylethylpropyl)acrylamide (HEAA), ammonium persulphate (APS), N,N′-methylenebisacrylamide (BIS), LiF, HCl (Conc.), and N,N,N′,N′-tetramethyl ethylenediamine (TMEDA) were purchased from Sigma-Aldrich (St. Louis, MO, USA) at the highest purity available and used without further purification. MiliQ water from an ultra-pure Milli Q water system from Direct-Q (Billerica, MA, USA) was used in all experiments and is denoted as DI water.

### 2.2. Preparation of the Ti_3_C_2_T_x_

Ti_3_C_2_T*_x_* was prepared using a protocol already published [44,45]. Concentrated HCl was diluted with distilled water to get 6 M solution (10 mL). 0.666 g LiF was added into the solution, with stirring for half an hour using a magnetic PTFE stir bar to dissolve the salt sufficiently. One gram of Ti_3_AlC_2_ powder was then added to the mixed solution gradually to avoid the violent exothermic reaction. The suspension was held at different temperature for 24 h. This step led to the creation of multilayered Ti_3_C_2_T*_x_* (ML-MXene), which was cleaned by extensive centrifugation (10 times) until the pH of the supernatant reaches a value over 4. The prepared ML-MXene was filtered through the membrane with the porosity 0.3 µm, washed with ethanol, and dried at 40 °C. Few layered Ti_3_C_2_T*_x_* (FL-MXene) was prepared from the multilayered Ti_3_C_2_T*_x_*. The exfoliated Ti_3_C_2_T*_x_* (0.4 g) were dispersed in distilled water (150 mL) and the delamination was performed by sonication via an inner probe sonicator probe with diameter of 7 mm (Hielscher Ultrasonics GmbH, Teltow, Germany) at an amplitude of 40%, cycle 0.5, and power density 300 W/cm^2^ for 20 min. Prepared FL-MXene suspension was centrifuged at 2000 rpm for 60 min.

Then, the supernatant containing the stable MXene colloidal solution was collected, vacuum filtrated through a polyethylene separator membrane (3501 Coated PP, Celgard, Charlette, NC, USA), and air-dried which led to obtaining dark powder of FL-MXene.

### 2.3. Preparation of MXene/NIPAm Hydrogel Nanocomposite

The typical MXene/NIPAm hydrogel-composite fabrication process was as follows. The MXene solution, with the amount of MXene shown in Table 1, was ultra-sonicated in a vial for 1 h in a laboratory sonicator bath at room temperature under a nitrogen atmosphere to ensure the complete dissolution of MXene. Subsequently, a degassed solution of 2 mmol NIPAm, 1 mL of aqueous MXene solution, 1 mol% BIS (crosslinker, molar to monomer), 40 µL of 0.22 M APS solution, and 14 µL of TMEDA were mixed thoroughly by vortex mixing for 1 min under a nitrogen atmosphere and transferred without delay between two glass plates 55 × 15 × 1 mm in dimension, with the thickness adjusted on the three sides using a 1 mm glass spacer. The resulting form was closed with a Parafilm tape on top and kept horizontally to facilitate the gelation process. After 6 h, the MXene/NIPAm gel slabs were removed from the form, washed thoroughly by immersion in a large amount of water, and water was exchanged several times to remove any unreacted monomers or additives.

### 2.4. Preparation of Bilayer MXene/NIPAm@HEAA Composite

Bilayer samples of MXene/NIPAm@HEAA were prepared in two steps. An initial layer of MXene/NIPAm (the so-called NIPAm layer) was prepared as stated above. After 10 min of gelation, the upper glass plate was removed, an additional glass spacer was added, and a pre-gelled solution of HEAA (2 mmol HEAA, 1 mol% BIS (crosslinker, molar to monomer), 40 µL of 0.22 M APS solution, and 14 µL of TMEDA) was laid over the first layer and covered by a glass plate. Both NIPAm and HEAA were of the same film thickness. The timing of the bilayer fabrication was adjusted such that at the time of pre-gel HEAA addition, NIPAm was in the state of a settling pre-gel. Ensuring that the NIPAm is a pre-gel improves the interfacial adhesion between the NIPAm and HEAA layers. After 12 h of aging, the bilayer films were removed from the form, and the bilayer slab was washed thoroughly with water in several cycles as a simple slab.

### 2.5. Swelling Determination

In this stage, 1 × 1 cm^2^ MXene/NIPAm specimens were cut with a razor and dried for 16 h. The weight of each sample was recorded as the dry weight (*W_d_*). Then, each specimen was immersed in DI water and stored in the dark for 6 h for the hydrogel to swell to equilibrium. Then, the hydrogel was gently wiped with a tissue to remove excess water, and the wet weight (*W_w_*) was measured in defined time intervals upon exposure to light for two minutes, followed by keeping the sample in the dark for 8 min.

The swelling ratio (*λ*) can be defined by Equation (1), as follows:

(1) $$\lambda =\frac{W_{w}-W_{d}}{W_{d}}$$

The swelling ratio was then converted into a normalized swelling ratio ([*λ*]) to make the comparison between different percentages of MXene in NIPAm more meaningful (Equation (2)). Here, *λ*_0_ is the swelling ratio at time *t* = *t*_0_:

(2) $$\left[\lambda \right]=\frac{\lambda }{\lambda_{0}}$$

### 2.6. Bilayer Actuation Performance Tests

The MXene/NIPAm@HEAA bilayers were dissected into thin strips with a much greater length than width. Using these dimensions, the interface of the bilayer at equilibrium in water remains perpendicular to the ground.

The shape effect was also studied using the 0.3MXene/NIPAm@HEAA sample. The dissected specimens were in rod and layered shapes.

The actuator performance test was carried out in the DI water medium, and the sample was initially allowed to reach its maximum swelling state in the dark. Then, the sample was exposed to either the white light of an Xe Arc lamp (70 mW cm^−2^), a white LED lamp (25 mW cm^−2^), or real sunlight (70 mW cm^−2^). The bending curvatures were assessed by photographs. Photographs were made in 20 s time intervals for up to 200 s with a Canon 80D camera (Tokyo, Japan). A reversibility test of the sample was performed by switching between light exposures and maintaining the samples in the dark to assess the reversibility in bending behavior.

### 2.7. Characterization

The XRD spectra were obtained by *θ* from 5 to 40° with Cu Kα radiation (Panalytical Empyrean, Almelo, Netherlands). The UV-Vis spectra of the samples were measured with a UV-Vis spectrometer in the range of Libra (Biochrom, Berlin, Germany). Fourier transform infrared (FTIR) spectra were obtained on a Frontier FTIR spectrometer (Perkin Elmer, Waltham, MA, USA) in ATR mode using a ZnSe crystal from 400 to 4000 cm^−1^. The SEM morphology was observed by scanning electron microscopy (SEM) (JCM 6000–Jeol microscope, Tokyo, Japan) at an accelerated voltage of 15 kV. The samples were then sputtered with a thin layer of gold.

## 3. Results and Discussion

### 3.1. Fabrication Design

**Figure 1:**
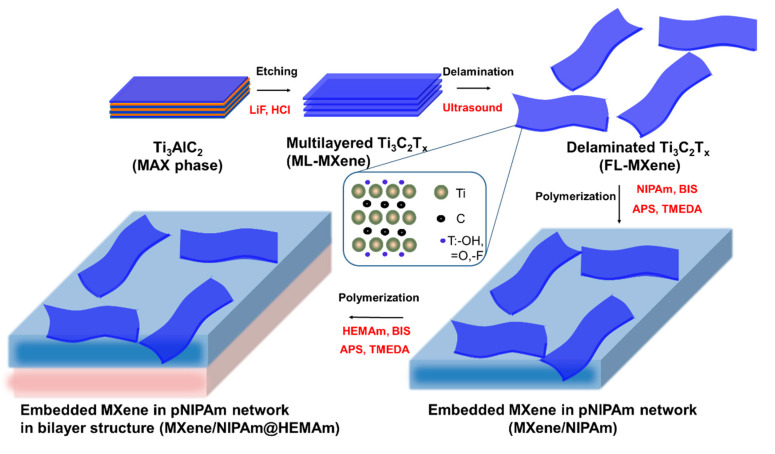
Schematic representation of MXene/NIPAm@HEAA bilayer formation.

The fabrication process for the MXene-based bilayer hydrogel formation is shown schematically in Figure 1. In the first step, the MAX phase of TI_3_C_2_Al was etched to prepare the multilayered MXene (ML-MXene). In the second step, delamination took place to obtain well-dispersed few layered MXene via ultrasound treatment. This nanomaterial was further used in the third step for the prepared NIPAm hydrogel layer together with the dispersed MXene as a filler. The fabrication process for the thermoresponsive layer formation of the well-dispersed FL-MXene was performed in a solution of NIPAm as the thermoresponsive part of the polymeric network, with BIS as a crosslinker. Free-radical polymerization was induced by APS and accelerated with TMEDA in the presence of an inert atmosphere. The polymerization reaction was performed under ambient temperatures to prevent any aggregation, phase separation, and/or degradation of the formed NIPAm-based hydrogel structure and MXene. In the final step, the second layer with an HEAA-based hydrogel was introduced to form the bilayer composite. In the bilayer hydrogel composite, the HEAA layer acted as the passive/support/buffer layer, and, generally, the HEAA layer was photothermally unresponsive, experienced no charges, and was neutral. Thus, any shrinking or swelling in the MXene/NIPAm layer resulted in bending of the overall bilayer hydrogel. The bilayer composite MXene/NIPAm@HEAA was fabricated by adding a gelation solution of HEAA with a crosslinker, initiator, and accelerator to the pre-gelled MXene/NIPAm layer under an inert nitrogen atmosphere. All vinyl-based components in the gelation process were chemically identical to the acrylamide-based compounds that minimize the composition drift in the polymerization crosslinking process [46].

### 3.2. MXene Filler Preparation and Characterization

**Figure 2:**
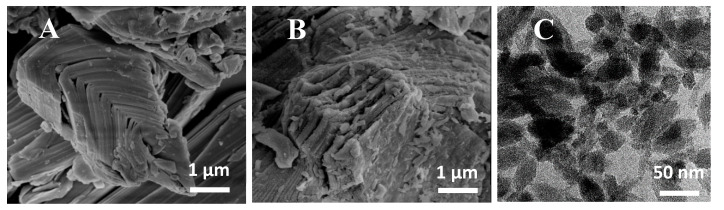
(**A**) SEM image of MAX-phase, (**B**) SEM image of exfoliated Ti_3_C_2_T*_x_* (ML-MXene), and (**C**) TEM image of delaminated Ti_3_C_2_T*_x_* (FL-MXene).

Ti_3_C_2_T*_x_* was prepared by etching Al from Ti_3_AlC_2_ (Figure 1) via the HF produced in situ through a reaction of LiF and HCl. This led to the exfoliation of the Ti_3_C_2_T*_x_* layers. Figure 2B shows SEM images of the exfoliated Ti_3_C_2_T*_x_*_._ Some defects and mild degradation are visible on the surface of the MXene flakes, which could be caused by LiF, the HCl etching solution [47], or the partial oxidation of Ti_3_C_2_T*_x_*. It was previously reported that MXenes are prone to degradation through their oxidation to TiO_2_ [48,49]. During preparation, only negligible oxidation was observed, with a peak at 27°. Subsequently, the exfoliated multilayered Ti_3_C_2_T*_x_* (ML-MXene) was delaminated by extensive sonication, and few Ti_3_C_2_T*_x_* (FL-MXene) layers were created, as seen in the TEM image (Figure 2C).

The main advantage of using LiF/HCl over HF was that both etching and intercalation proceeded in one step during the exfoliation process, which significantly simplified the delamination of Ti_3_C_2_T*_x_* [44].

**Figure 3:**
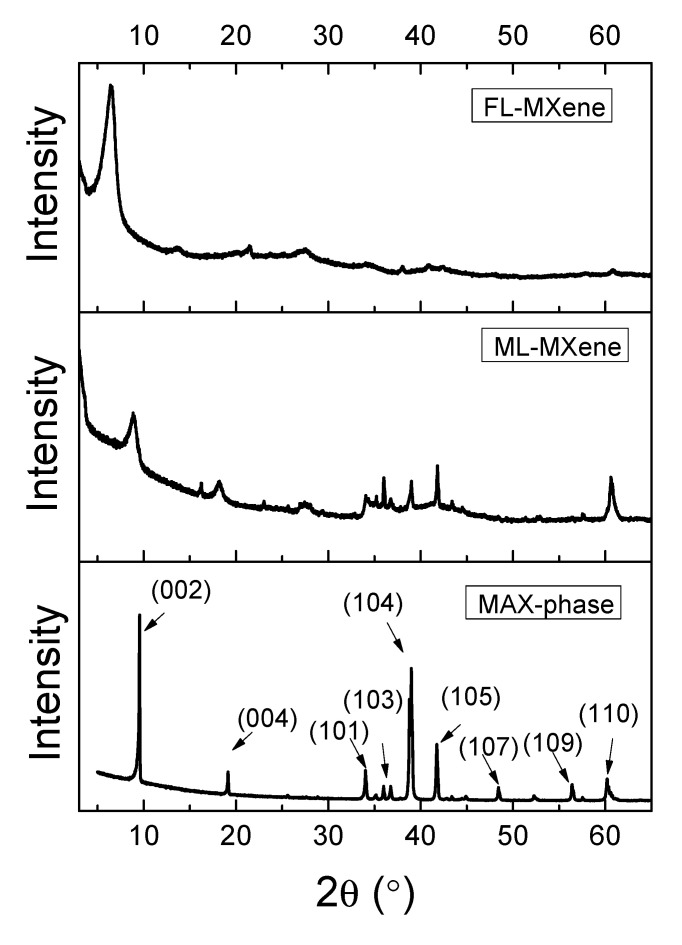
XRD diffractogram of FL-MXene, ML-MXene, and MAX-phase films.

XRD analysis confirmed the removal of Al atoms from the MXene layers of Ti_3_AlC_2_ (Figure 3). Broadening and downshifting of the peak (002) of Ti_3_AlC_2_ at 9.5° was observed for ML-MXene at 7.8° and FL-MXene at 6.5°, which indicated an expansion of the structure and larger d-spacing between 2D MXene layers and water molecules intercalation. In addition, the non-basal plane peak from the MAX phase (the peak at 39°) decreased in intensity for ML-Ti_3_C_2_T*_x_* and even disappeared for FL-Ti_3_C_2_T*_x_*. Furthermore, we observed broadening, decreases in intensity, and/or shifts in the peaks of (002), (004), and (101), (103), (105), (107), (109), and (110) after the etching process compared to MAX phase. This result is mainly due to the removal of Al from the Ti_3_AlC_2_ [45].

### 3.3. MXene/NIPAm Characterization

**Figure 4:**
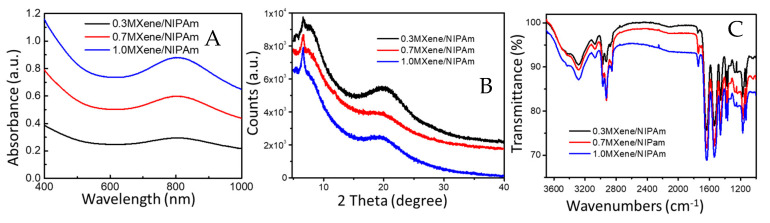
(**A**) UV-vis-NIR absorbance spectra; (**B**) XRD diffractograms; and (**C**) FTIR spectra of 0.3 (black line), 0.7 (red line), and 1.0MXene/NIPAm (blue line) samples.

Three different concentrations of MXene were chosen and resulted in 0.3, 0.7, and 1.0MXene/NIPAm samples. The successful incorporation and concentration in a hydrogel polymeric network were examined by UV-Vis spectroscopy (Figure 4A). The UV-Vis spectra showed a gradual increase in absorption at 800 nm corresponding to MXene absorption and successful incorporation into the polymer network. The exact concentration can be estimated based on the calibration curve of the solution (0.33, 0.67, and 1.04% mg/mL of hydrogel) after extracting the spectra of NIPAm. The polymeric network embedded MXene and negligible leakage (approx. 2%) were observed after water immersion following preparation. These results are attributable to the presence of MXene on the surface or in proximity to the interphase, and no further leakage was observed in water immersion after several cycles of water exchange.

For characterization purposes, different MXene/NIPAm composites were prepared and freeze-dried for 48 h to obtain the relevant xerogels of MXene/NIPAm. XRD measurements were carried out to determine that MXene was incorporated into the polymeric network in a dispersed form, as depicted in Figure 4B. The XRD showed a broad peak at 6° that is attributed to the (002) peaks of Ti_3_C_2_ MXene and lower values when prepared with MXene, with a continual increase from 0.3 to 1% MXene/NIPAm. These observations confirm the increase in interlayer spacing, which is also related to the delamination and successful incorporation of MXene’s structure in the polymeric network. Moreover, the signals at *θ* = 9.39° and 2*θ* = 19.50° with very wide diffraction patterns were attributed to the amorphous and very low crystallinity of the p(NIPAm) polymeric network structure.

The FTIR spectra in Figure 4C showed the main functional groups of NIPAm with their absorption peaks at 3200, 2800, 1620, 1540, and 1500 cm^−1^, which are attributed to N–H stretching vibrations, C–H stretching vibrations, C=O amide I, C=O amide II stretching vibrations, and C–H bending vibrations, respectively. The C=O absorption peak from MXenewas indicated at 1650 cm^−1^, and the peak increases with an increase in the MXene incorporated into the samples.

**Figure 5:**
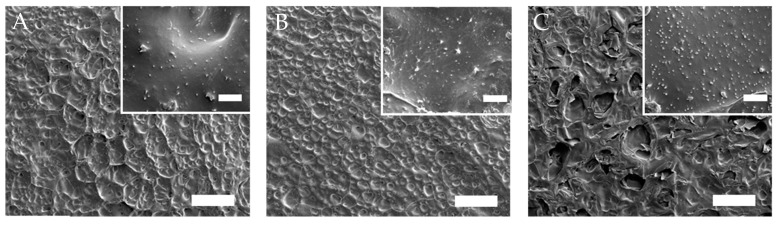
SEM images of the xerogels of (**A**) 0.3, (**B**) 0.7, and (**C**) 1.0MXene/NIPAm samples; the bar scale indicates 200 µm (inset images: bar scale 10 µm).

SEM was used to further investigate the morphology of the sample (Figure 5). SEM revealed that all samples had a three-dimensional network with homogenous and microporous structures evenly distributed in the samples. Pores ranged from tens to hundreds of micrometers in size with dense walls that agree with those of similar conventional NIPAm–based hydrogels [50]. Images at a higher magnification showed uniformly distributed particles in a porous structure. The concentration of particles observed in the hydrogel structure increased by increasing of MXene in the feed. These observations indicate the homogenous distribution of the MXene structure in the polymer network of the composite structures.

**Figure 6:**
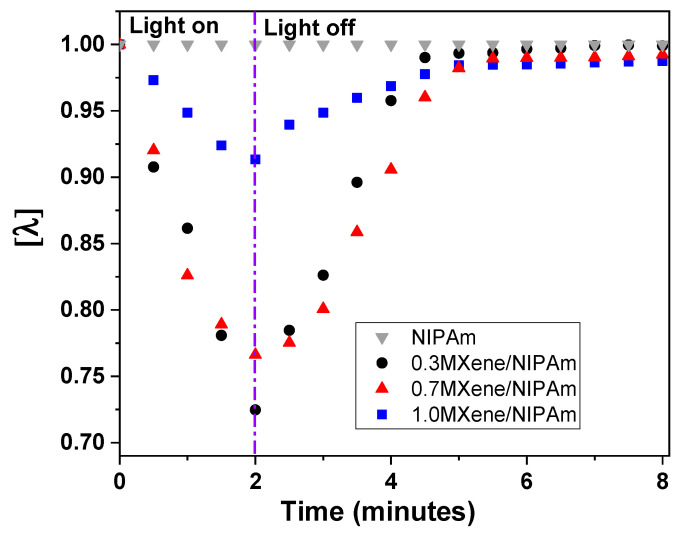
Normalized swelling ratio [*λ*] of the MXene/NIPAm samples. Light was exposed for 2 min to MXene/NIPAm slab samples in an equilibrium state followed by continuing in the light off regime until swelling back to the equilibrium state (light on–off separation is also denoted by vertical dashed line).

The swelling ratio (*λ*) of the light-responsive hydrogel is described as the extent to which the hydrogel remains in or swells from its equilibrium state upon exposure to light or dark, respectively. The swelling ratio was measured for MXene/NIPAm samples with different MXene content. As seen in Figure 6, the 0.3MXene/NIPAm sample has the highest normalized swelling ratio among the MXene/NIPAm samples. MXene adds structural integrity to the hydrogel network structure. However, increasing the MXene content limits the swelling degree of the hydrogel. Additionally, the degree of swelling is an indicator of the sensitivity of the hydrogel material’s performance as an actuator. The swelling ratio of the MXene/NIPAm layer is an important parameter (Figure 6) to describe the swelling behavior and, consequently, the actuator performance of this NIPAm layer. The control sample with the hydrogel from NIPAm clearly shows the photothermal effect of MXene. The sample after irradiation did not show any changes in swelling or shrinking after the experiment.

The actuation time constant (*τ*) was calculated for the bending and relaxation of the thin strip actuator materials with different MXene content by fitting data into the following equations:

For bending:

(3) $$\alpha =\alpha_{0\;}\left[1-\;e^{-\left(t/\tau \right)}\right]$$

For relaxation:

(4) $$\alpha =\alpha_{0\;}[e^{-\left(t/\tau \right)}]$$

where *α*_0_ refers to the maximum bending angle, and *t* represents the time at which bending angle *α* is measured [51]. The extracted actuation time constant was 74 s for 0.3MXene/NIPAm but was slightly higher at 90 s for 0.7MXene/NIPAm and 1.0MXene/NIPAm. This indicates that the fastest swelling changes and most efficient performance belong to the 0.3MXene/NIPAm sample. Further discussion about the influence of MXene loading on the swelling properties is provided below.

### 3.4. MXene/NIPAm@HEAA Bilayer Hydrogel Performance

**Figure 7:**
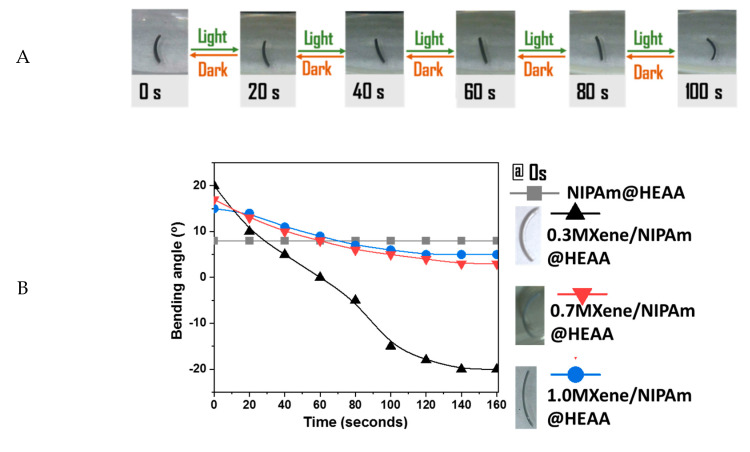
(**A**) micrographs of the 0.3MXene/NIPAm@HEAA bilayer samples taken in 20 s intervals under visible light irradiation, light intensity 25 mW cm^−2^. (**B**) Bending curvature vs. time graph for the bilayer MXene/NIPAm@HEAA samples with different MXene content. Lower insets show the schematic bending angle *α* for determination of the bilayer actuator. Micrographs to the right show the bilayer samples at the beginning of the experiment.

The bending behavior of the bilayer hydrogels with all three MXene concentrations was studied under visible light irradiation at a 25 mW cm^−2^ light intensity using a white LED lamp. For this purpose, the bilayer hydrogel was cut into a thin strip-like shape, and the reversibility of the shape was also evaluated by discontinuing the application of light, as depicted in Figure 7A. The bending behavior of the strips was further analyzed in terms of changes in the bending angle over time and plotted in Figure 7B. The bending angle was measured by drawing a tangent passing through the arc at the midpoint of the bilayer sample. Curvature was taken as the angle between the tangent and the boundary of the outer layer. Angle *α* was considered positive if the outer layer was NIPAm, whereas the angle was deemed to be negative for the HEAA outer layer, as shown in the Figure 7B inset. The 0.3MXene/NIPAm@HEAA bilayer sample demonstrated a more promising sensitivity and response rate than the 0.7MXene/NIPAm@HEAA and 1.0MXene/NIPAm@HEAA samples. Before the bending performance tests, strips were kept in fresh DI water in the dark to ensure that the NIPAm layer was equilibrated with the maximum absorbable amount of water at ambient temperatures below the LCST of NIPAm. Since the length is much larger than the width of the strips, the center of gravity keeps the strips flipped such that the interface between the two layers is perpendicular to the ground. As a result, the bilayer looks like the letter “C,” where the outer layer of the “C-shape” is NIPAm. With the lights on, the NIPAm part of the strip gradually absorb light and photothermally shrink the NIPAm layer. The resulting volume change occurring with respect to the immobile HEAA layer causes the overall bilayer to bend outwardly. This makes the strip transition from a “C-shape” to an “I-shape,” followed by inverted “C-shape.” The contribution of MXene in the photothermal behavior of MXene/NIPAm is crucial, as the sample without MXene did not show any bending changes during the experiment. MXene is known for its high heat conductivity [52]. This increases the sensitivity/efficiency of the photothermal process. However, according to the observations in Figure 4B, increasing the MXene content above 0.34% did not improve bending performance. In fact, the change in curvature after 140 s for 0.34% MXene was 200%, whereas the curvature change for both 0.7% and 1.0% MXene lay between 50% and 60%. This surprisingly low actuator sensitivity with increased MXene content could be an outcome of the distribution of NIPAm and MXene on the exposed facet. More MXene, above a certain amount, apparently hinders the free motion of the NIPAm network for shrinking and swelling. Even though MXene shows a high heat capacity, these inorganic pebbles in the NIPAm network act as junctions or halt points, limiting the movement of NIPAm chains. Ti^2+^ is centered in the top layers of MXene when in excess, thereby making physical crosslinks with lone pairs of electrons in the “N” of NIPAm. Similarly, in a nitrile polymer and Zn^2+^ salt-based hydrogel actuator system, Bai et al. observed Zn^2+^ salt to limit its relaxation when the Zn^2+^ salts in the solution reach saturation due to the physical crosslinks made between the nitrile and Zn^2+^, where the two nitrile groups bind to a single Zn^2+^ center [53]. The temperature and pH do not influence this process directly; however, they affect the mixing entropy, which in turn controls the free energy of mixing [13]. The swelling degree depends on internal factors like crosslinking density, as well as external factors such as the temperature and the medium’s pH. For different systems with different NIPAm additions, the temperature and pH remain consistent while the crosslinking density changes regardless of the fact that the BIS, the polymer crosslinker in the feed, was the same for all compositions. Hence, the 0.3MXene/NIPAm@HEAA sample offers a favorable balance in the distribution of NIPAm and MXene in the NIPAm monolayer, leading to synergistic performance.

**Figure 8:**
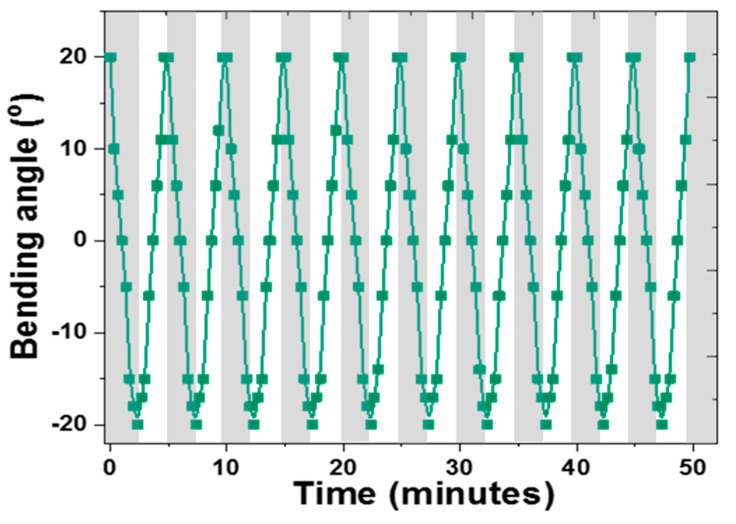
Bending curvature vs. time graph for the cycling behavior of the 0.3MXene-/NIPAm)@HEAA bilayer over ten consecutive light on (grey region) and light off (white region) cycles.

The reversibility of the actuator performance with and without illumination, over several light on–off cycles, is important for assessing the feasibility of practical implementation. For this reason, a thin strip of 0.3MXene/NIPAm@HEAA was subjected to ten consecutive light on–off cycles for a 50 min duration, and the repeatability was excellent over all ten cycles (Figure 8). Kim et al. observed similar cycling behavior in the NIPAm/graphene oxide system [22] without significant changes in the actuator time scale. In the g-PNIPAm/MNP system, the maximum curvature showed slight variations over 30 cycles under identical illumination conditions [23], whereas in our system, the curvature and time scale were reproduced very well over the ten cycles studied.

### 3.5. Effect of the Shape of the Bilayer Actuator and Light Intensity on Actuation Performance

**Figure 9:**
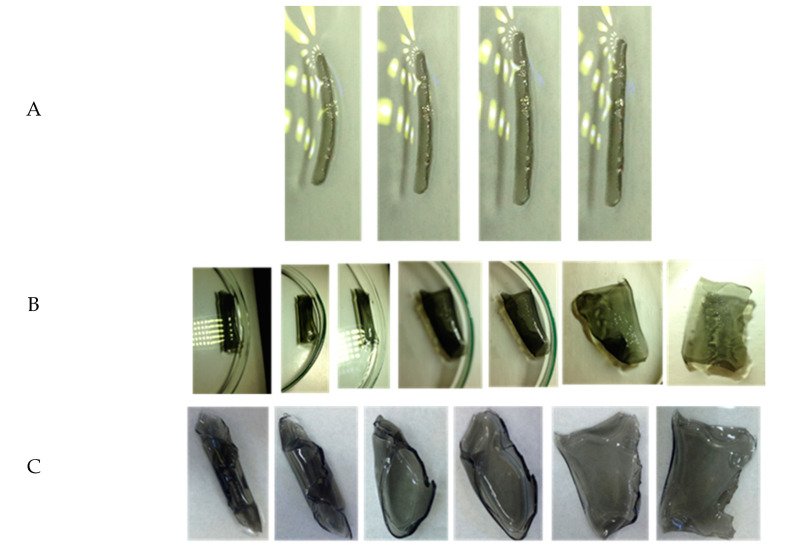
Actuator micrographs taken at a 20 s time interval for (**A**) a rectangular rod (LED) and (**B**) and (**C**) a layer shaped film under LED light and Xe lamp exposure, respectively.

It is known that deformation is different for differently shaped stimuli-responsive specimens. In this study, apart from thin strips, we evaluated the actuator behavior of a rod and layer-like shape. For both shapes, where the length and width dimensions are similar, an interlayer of the bilayer is parallel to the ground. The rod, upon exposure to white LED light (25 mW cm^−2^), moved inward from the NIPAm side until it settled in a straight orientation after being curved (Figure 9A), similar to the behavior of the thin strip. Complementary inward bending was observed with the reduced graphene oxide-PNIPAm/polyacrylamide bilayer strip, with PNIPAm comprising the outer layer and entering the inner layer with exposure to light [22]. In contrast to the thin strips and the rod, the layered specimen in a swollen state (in DI water kept in the dark) curled along the diagonal axis, as can be seen in Figure 9B, with NIPAm in the bottom layer. Diagonal rolling was commonly observed when the aspect ratio of the layer was closer to that of a square (for *L*/*W* = 1 and *L*/*W* >1: *L*–length, *W*–width) [54]. Generally, the layers of bilayer hydrogels in a swollen state curled along the short axis (longitudinal), long axis (transversal), or the diagonal. The combined effects of the aspect ratios of the cut layers and the homogeneity of each layer were found to govern the exact direction of curling [55,56]. The diagonally curled NIPAm/HEAA layer with exposure to white LED light (25 mW cm^−2^) started to uncurl gradually until the sample became completely flat.

The sample was also studied for illumination under white light from an Xe Arc lamp (70 mW cm^−2^), as depicted in Figure 9C. The uncurling time scale in an entirely flat position was faster for the Xe lamp compared to the white LED. This is expected for two reasons. First, the light intensity is low under a white LED; therefore, the amount of photon energy absorbed by the MXene units is low, resulting in a slower degree of actuation. Second, along with the light, heat is emitted and reaches the sample. This heat, undoubtedly, also contributes to the photothermal behavior of NIPAm@HEAA bilayer films [57]. This emission of secondary heat is lower under LED light than under the Xe lamp. The net effect is a slight drop in the actuator performance rate under LED light illumination. The high thermal conductivity of MXene, however, compensates for the lower light intensity of the LED lamp by efficiently transferring heat to produce light-induced drying in the NIPAm layer and managing to reduce the delay on a time scale to only 20 s. Regardless of the complex actuator performance in the layers compared to that of the thin strips or rods, the extent of bending or curling remained proportional to the intensity of the incident light in the bilayer polymorphs [13,57]. Notably, during these experiments, the structural integrity exhibited between the passive HEAA and light-responsive NIPAm layer was promising for use in real applications. However, a one to one comparison of similar actuator systems remains difficult. In most cases, the actuator strip is fixed on one end, and the actuation on other end is monitored. The lack of uniformity in sample selection and data output methods also creates problems for such analyses.

**Figure 10:**
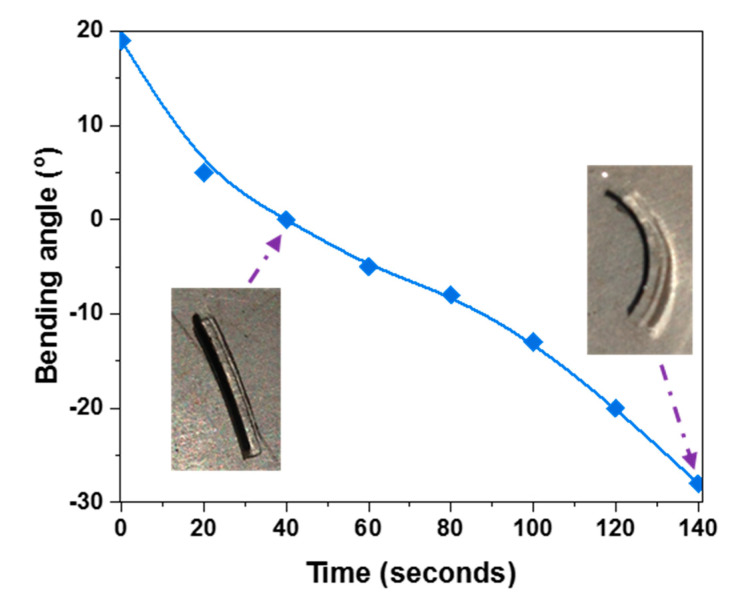
Bending angle vs. time graph for the thin 0.3MXene/NIPAm@HEAA strip under real sunlight with a 70 mW cm^−2^ intensity illumination.

A specimen of a thin 0.3MXene/NIPAm@HEAA strip was held under real sunlight at an intensity of 70 mW cm^−2^ over two minutes to examine the degree of bending under sunlight. This feature is important for evaluating the potential of the prepared NIPAm@HEAA bilayers in sensor designs to help the blind recognize the sunlight. The bending angle change over time, shown in Figure 10, demonstrates outstanding bending curvature changes upon solar illumination. Comparing the actuation rate with that under white LED light irradiation at a 25 mW cm^−2^ intensity (Figure 7B) clearly shows that the actuation is faster upon exposure to real sunlight. In both scenarios, the thin strip under equilibrium in the dark had a bending angle of 20° with 0.3MXene/NIPAm providing the outer layer. It achieved zero curvature after 60 s under LED illumination, whereas the time reduced to 40 s under real sunlight. Furthermore, after 140 s of illumination, the thin strip held under the LED light showed 20° bending in the curvature where the HEAA made up the outer layer, while the curvature was greater at 28° for the thin strip kept under sunlight. The numbers indicate a 14% reduction in the time scale of the actuator behavior under sunlight at an intensity lower than 1 sun (100 mW cm^−2^). This faster actuation in the sunlight is favorable, as MXene/NIPAm@HEAA is expected to act as a sensor for sunlight. The faster actuation that we observed could be related to the wavelength profile of the emitted light.

## 4. Conclusions

We successfully fabricated a MXene/NIPAm@HEAA bilayer composite with three MXene loading concentrations of 0.3, 0.7, and 1%. Ti_3_C_2_T*_x_* MXene was incorporated into the MXene/NIPAm hydrogel structure by free radical polymerization and characterized by XRD, FTIR, UV-Vis spectrometry, and SEM. The photothermal behavior of the MXene/NIPAm hydrogel samples was elucidated by the changes in swelling upon light irradiation, which revealed that the actuation time constant was as low as 74 s for the 0.3MXene/NIPAm sample. Moreover, the photothermally induced swelling changes were reproducible and repeatable over ten consecutive cycles of a light on and off regime. The bilayer actuator of 0.3MXene/NIPAm@HEAA showed the highest bending curvature change (200%) after 100 s of white light exposure at a 70 mW cm^−2^ intensity. The shape effect and light effect were also evaluated with respect to the deformation shape and light. The suitability and implementation in solar light sensing applications were examined by monitoring the bending curvature of the thin strip sample held in real sunlight. Such spatially and remotely light-control behavior opens avenues for applications in microfluidic and light detection technologies.

## Figures and Tables

**Table 1:** Amount of the component combinations used in the MXene/NIPAm and MXene/NIPAm@HEAA-based samples.

Codesample	NIPAm (mg)	HEAA(mg)	MXene (mg)	BIS (mg)	DI (mL)	0.22 M APS (μL)	TMEDA (μL)
0.3MXene/NIPAm	226	-	8	4	2	80	28
0.7MXene/NIPAm	226	-	16	4	2	80	28
1.0MXene/NIPAm	226	-	24	4	2	80	28
0.3MXene/NIPAm@HEAA	226	475	8	8	4	240	56
0.7MXene/NIPAm@HEAA	226	475	16	8	4	240	56
1.0MXene/NIPAm@HEAA	226	475	24	8	4	240	56
